# Association of Childhood Externalizing, Internalizing, and Comorbid Symptoms With Long-term Economic and Social Outcomes

**DOI:** 10.1001/jamanetworkopen.2022.49568

**Published:** 2023-01-09

**Authors:** Francis Vergunst, Melissa Commisso, Marie-Claude Geoffroy, Caroline Temcheff, Martine Poirier, Jungwee Park, Frank Vitaro, Richard Tremblay, Sylvana Côté, Massimilliano Orri

**Affiliations:** 1 Department of Special Needs Education, University of Oslo, Oslo, Norway; 2 Department of Social and Preventive Medicine, University of Montreal, Montreal, Québec, Canada; 3 Ste-Justine University Hospital Research Center, Montreal, Québec, Canada; 4 Department of Psychology, Concordia University, Montreal, Québec, Canada; 5 McGill Group for Suicide Studies, Department of Psychiatry, Douglas Mental Health University Institute, Montreal, Québec, Canada; 6 Department of Educational and Counselling Psychology, McGill University, Montreal, Québec, Canada; 7 Department of Education, University of Rimouski, Rimouski, Québec, Canada; 8 Statistics Canada, Ottawa, Ontario, Canada; 9 Department of Psychoeducation, University of Montreal, Montreal, Québec, Canada; 10 Department of Psychology, University of Montreal, Montreal, Québec, Canada; 11 Bordeaux Population Health Research Centre, INSERM U1219, University of Bordeaux, Bordeaux, France

## Abstract

**Importance:**

Little is known about the long-term economic and social outcomes for children with longitudinally assessed comorbid externalizing and internalizing symptoms, especially compared with children with externalizing symptoms or internalizing symptoms only.

**Objective:**

To examine the association between childhood trajectories of externalizing, internalizing, and comorbid symptoms and long-term economic and social outcomes.

**Design, Setting, and Participants:**

A 32-year prospective cohort study linked with administrative data was conducted in school-aged participants aged 6 to 12 years in the Québec Longitudinal Study of Kindergarten Children (N = 3017) followed up from 1985 to 2017. Data analysis was conducted between August 1, 2021, and March 31, 2022.

**Exposures:**

Teacher-rated behavioral symptoms were used to categorize children from age 6 to 12 years into developmental profiles using group-based trajectory modeling.

**Main Outcomes and Measures:**

Multivariable regression models were used to test the association between childhood symptom profile group and adult employment earnings, welfare receipt, intimate partnership status, and having children living in the household. Participant sex, IQ, and socioeconomic background were adjusted for.

**Results:**

Of 3017 participants in this sample, 1594 (52.8%) were male and 1423 (47.2%) were female. Per confidentiality rules established by Statistics Canada, income variables were rounded to base 100 and count variables were rounded to base 10; the mean (SD) age was 37 (0.29) years at follow-up. Four symptom profiles were identified: no/low (n = 1369 [45.4%]), high externalizing (882 [29.2%]), high internalizing (354 [11.7%]), and comorbid (412 [13.7%]) symptoms. Compared with the no/low symptom profile, participants in the high externalizing–only profile earned $5904 (95% CI, −$7988 to −$3821) less per year and had 2.0 (95% CI, 1.58-2.53) times higher incidence of welfare receipt, while participants in the high internalizing group earned $8473 (95% CI, −$11 228 to −$5717) less per year, had a 2.07 (95% CI, 1.51-2.83) higher incidence of welfare receipt, and had a lower incidence of intimate partnership (incident rate ratio [IRR], 0.89; 95% CI, 0.80-0.99). Participants in the comorbid profile fared especially poorly: they earned $15 031 (95% CI, −$18 030 to −$12 031) less per year, had a 3.79 (95% CI, 2.75-5.23) times higher incidence of annual welfare receipt, and were less likely to have an intimate partner (IRR, 0.71; 95% CI, 0.63-0.79) and children living in the household (IRR, 0.86; 95% CI, 0.80-0.92). Estimated lost earnings over a 40-year working career were $140 515 for the high externalizing, $201 657 for the high internalizing, and $357 737 for the comorbid profiles.

**Conclusions and Relevance:**

In this cohort study, children exhibiting sustained childhood high externalizing, high internalizing, or comorbid symptoms were at increased risk of poor economic and social outcomes into middle age. These findings suggest that children exhibiting comorbid problems were especially vulnerable and that early detection and support are indicated.

## Key Points

**Question:**

What are the long-term economic and social outcomes for children with high externalizing, high internalizing, and comorbid externalizing and internalizing symptoms?

**Findings:**

In this cohort study including 3017 children, those who exhibited ongoing high externalizing, high internalizing, or comorbid symptoms had poor economic and social outcomes from age 18 to 37 years, including lower employment earnings and a higher incidence of welfare receipt. Children with comorbid externalizing and internalizing symptoms fared particularly poorly.

**Meaning:**

The findings of this study suggest that children exhibiting chronically elevated externalizing, internalizing, or comorbid externalizing and internalizing symptoms are vulnerable to long-term economic and social exclusion and that early detection and prevention are indicated.

## Teaser

This cohort study examines the outcomes of children with externalizing, internalizing, and comorbid externalizing and internalizing symptoms over a 32-year follow-up period.

## Introduction

Behavior and emotional problems are common among school-aged children and frequently co-occur.^1^ Studies of the statistical structure of these underlying symptom presentations noted that they are characterized by 2 broad factors: externalizing and internalizing problems.^2,3^ Externalizing problems include traits such as hyperactivity, impulsivity, aggression, and rule violation, while internalizing problems are characterized by worry, anxiety, depression, and social withdrawal.^4^ Long-term studies reported that children who exhibit externalizing or internalizing problems are more likely to experience future unemployment, criminal convictions, lower earnings, welfare receipt, intimate partnering difficulties, poor health, and earlier mortality.^5,6,7,8,9^ However, little is known about the economic and social outcomes of children with co-occurring externalizing and internalizing problems, especially compared with those in children with externalizing or internalizing problems only, which the present study sought to address.

Prevalence estimates for children with comorbid externalizing and internalizing symptoms vary across study populations and jurisdictions from 2.4% in a British sample followed up from childhood to adolescence^10^ to 13.7% from age 6 to 12 years in the Canadian cohort used in the present study^11^ to 17.8% to 34.4% in a cross-sectional community sample of kindergarten through 12th-grade students in 4 US states.^12^ While evidence for sex differences in the prevalence of childhood comorbid externalizing and internalizing problems is mixed,^11,13,14,15,16^ outcome studies note that both males and females fare poorly across a plurality of functional and educational domains.

Compared with those without externalizing or internalizing problems, children who exhibit comorbid symptoms are more likely to experience teacher-child relationship difficulties and lower math and reading performance through grades 1 to 12^17,18^; depressive, psychotic, and borderline personality disorder symptoms at age 11 to 12 years^19^; peer difficulties and increased risk-taking behavior^20^; harmful substance use and violence by late adolescence^21,22^; delinquency and social exclusion at age 20 years^23^; and self-harm and suicide by early adulthood.^11,24^ So far, very little is known about the association between childhood comorbid externalizing and internalizing symptoms and long-term economic and social outcomes, especially relative to those with childhood externalizing or internalizing symptoms only. Previous work found that clinical severity increases as the number of comorbid diagnoses increases,^25,26^ and children with comorbid symptoms are therefore likely to have worse social and economic outcomes than children who exhibit problems in one symptom category only. If confirmed, this result would have implications for early detection and prevention for this highly vulnerable subpopulation.

The present study draws on a population-based birth cohort (N = 3017) to examine the association between previously created childhood behavioral symptom profiles from age 6 to 12 years (no/low symptoms, high externalizing symptoms only, high internalizing symptoms only, comorbid symptoms) and economic and social outcomes from age 19 to 37 years, defined as employment earnings, welfare receipt, intimate partnership, and having children living in the household. International surveys report that these economic and social outcomes are consistently ranked as important and desirable in the lives of younger people.^27^ Furthermore, stronger personal financial circumstances are robustly associated with greater subjective well-being, better health, and a longer life.^28,29,30^ Intimate partnership is linked with stronger social support networks, higher well-being, and better health behaviors—with these associations likely to be at least partially causal^31,32^—while parenting responsibilities can increase personal meaning^33^ and shape the health, education, and personal values of subsequent generations.^34^ In the present study, it was expected that participants in the childhood comorbid symptom profile would fare poorly on all outcomes, compared with all other symptom profiles, while participants in the high externalizing-only or high internalizing-only profiles would have worse outcomes than participants in the no/low symptom profile. Since sex is known to be associated with the prevalence and distribution of childhood emotional and behavioral problems and with economic and social outcomes in adulthood, we examined sex as both a main effect and a moderator of the association between childhood behavior profile and adult life outcomes.

## Methods

### Participants

Data were drawn from the Québec Longitudinal Study of Kindergarten Children, a population-based birth cohort of children (N = 3017 [1594 males, 1423 females]) recruited in 1986-1987 and 1987-1988 while attending kindergarten.^35^ The sample comprised 2000 children (1001 boys) who were selected at random and 1017 children (593 boys) who scored at or above the 80th percentile for disruptive behavior problems on completion of kindergarten (age 5 or 6 years) using sex-specific cutoffs. The children’s parents provided written informed consent prior to participation; participants received financial compensation. The study was approved by the ethics boards of the University of Montreal, McGill University, and Statistics Canada. Data analysis was conducted between August 1, 2021, and March 31, 2022. This study followed the Strengthening the Reporting of Observational Studies in Epidemiology (STROBE) reporting guideline.

### Economic and Social Outcomes

Outcome data were obtained from participants’ tax return records from 1998 to 2017.^36^ Records included details about personal earnings, welfare receipt, intimate partnership status, and children living in the household for each year of follow-up. Employment earnings were defined as all pretax wages, salaries, and commissions, not including income from capital gains. Earnings were averaged for the 5 most recent years (2013-2017) and treated as a continuous outcome; earnings at or above the 99th income percentile were winsorized to remove extreme outliers. All earnings and income variables were converted to US dollars using 2017 purchasing power parity ($1 US = $0.83 CA). Social assistance support (welfare) is provided by the Canadian government as last resort financial support for people with insufficient income who are ineligible for unemployment insurance, excluding those who are incapable of work.^37^ For each participant, scores were calculated annually (received = 1, not received = 0) and summed across follow-up (1998-2017) to create a count variable.

Partnership status was defined as being in a conjugal relationship vs not (married/cohabiting = 1, single/separated/divorced/widowed = 0), during each year of follow-up from 1998 to 2017, with scores summed to create a count variable. According to Canada Revenue Agency regulations, individuals who are in a conjugal relationship (ie, legally married or cohabiting for 12 consecutive months or more) are required to report this status when filing their tax return.^38^ A conjugal relationship is “one of some permanence, when individuals are interdependent—financially, socially, emotionally and physically—when they share household and related responsibilities, and when they have made a serious commitment to one another.”^39^ Children in the household was defined as the number of years in which the participant had 1 or more children living in the household (eg, younger sibling, biological child, and stepchild) across follow-up. Scores for each year (yes = 1, no = 0) were summed across follow-up (1998-2017) and treated as a continuous variable.

### Behavior Assessment

Behavioral symptoms were assessed annually by the children’s school teachers using the Social Behavior Questionnaire from age 6 to 12 years.^40^ The Social Behavior Questionnaire is widely used and has good psychometric properties and predictive validity across a range of outcomes.^41^ Externalizing problems were assessed using 13 items, eg, fights with other children; bullies/intimidates other children; and is squirmy, fidgety (α scores = 0.89-0.93). Internalizing problems were assessed using 5 items, eg, appears to be miserable, unhappy, or distressed; fearful or afraid of new things or situations; and cries easily (α scores = 0.61-0.76). The frequency of each behavior was rated on a 3-point scale as follows: never/not true = 0, sometimes/somewhat true = 1, and often/very true = 2. For each year, a confirmatory factor analysis was used to derive the latent externalizing and internalizing scores. Details of the approach, including a full list of included Social Behavior Questionnaire items and the final best-fitting model, are described elsewhere.^11^

### Symptom Profiles

**Figure 1.**
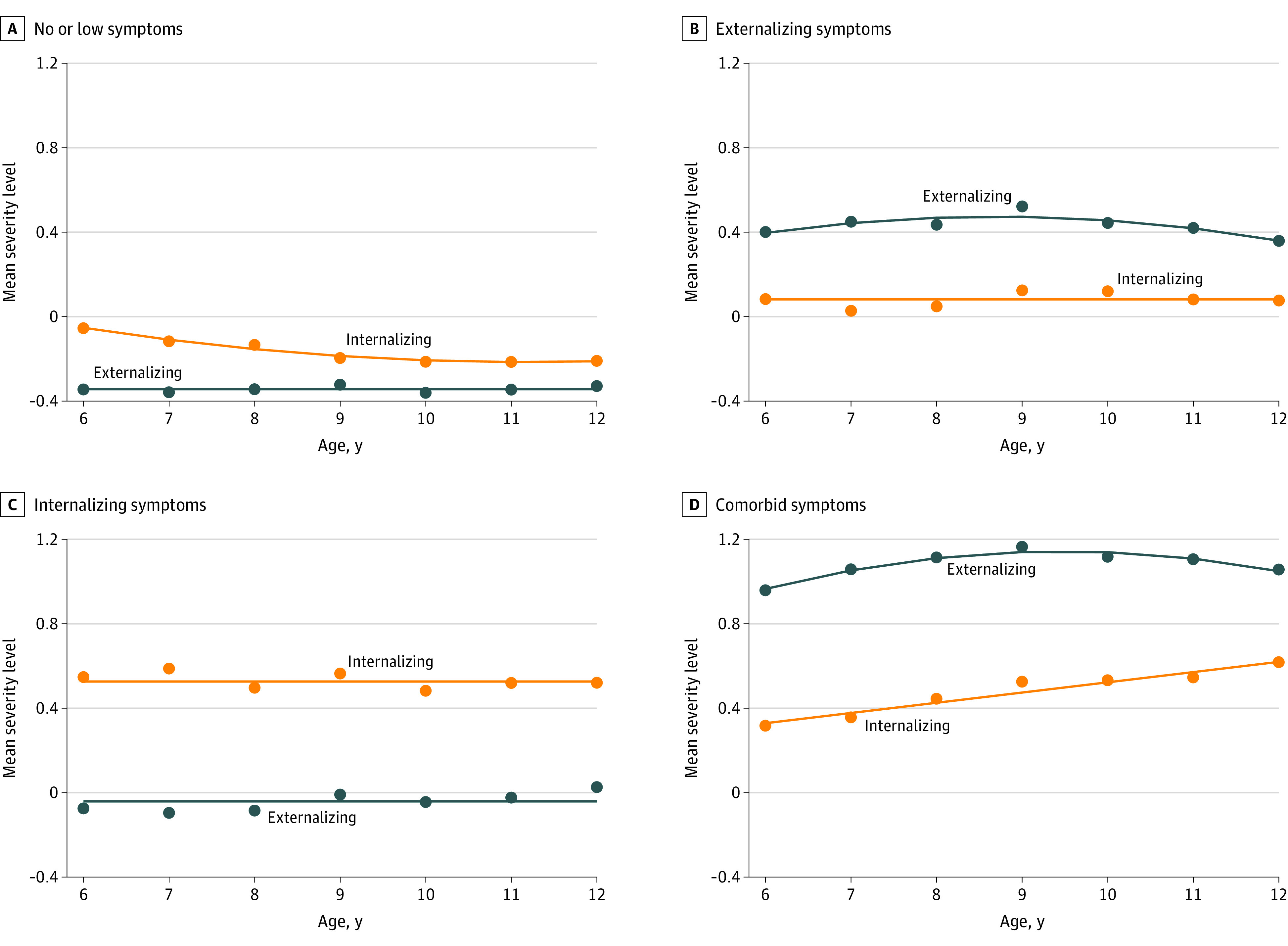
Trajectories of Childhood Externalizing, Internalizing, and Comorbid Symptoms The y-axis shows participants’ teacher-rated behavioral problems on the latent internalizing and externalizing factor scores derived from confirmatory factor analysis.^1^ Dots show the observed data points, and lines show the expected trajectories. Trajectories shown in individuals with no/low (1369 [45.4%]) (A), high externalizing symptoms (882 [29.2%]) (B), high internalizing symptoms (354 [11.7%]) (C), and comorbid symptoms (412 [13.7%]) (D). Adapted from Commisso et al.^1^

The derived externalizing and internalizing scores from age 6 to 12 years were used to create symptom profiles using group-based multitrajectory modeling. This statistical approach, based on mixture modeling and maximum likelihood estimation, can be used to identify groups (profiles) of children following similar symptom trajectories across time on multiple behavioral dimensions (in this case, externalizing, internalizing, and comorbid symptoms).^42^ Four distinct profiles were identified (Figure 1): no/low symptoms (1369 [45.4%]), high externalizing symptoms (882 [29.2%]), high internalizing symptoms (354 [11.7%]), and comorbid symptoms characterized by high externalizing symptoms and high internalizing symptoms (412 [13.7%]). Model invariance statistics are reported in eTable 1 in the Supplement.

### Control Variables

The following variables, which are associated with emotional and behavioral problems in childhood and economic and social outcomes in adulthood, were measured when participants were aged 6 years and adjusted for in the analyses. The child’s sex, the parents’ age at birth of their first child, parents’ total years of education, family structure (intact/2 parent = 0, nonintact/single parent = 1), and the parents’ household income (obtained from tax return records for the period 1984-1986). The child’s verbal IQ was adjusted for using the Sentence Completion Task administered at age 13 years.^43^ The instrument correlates highly with other measures of verbal and nonverbal IQ and educational attainment across respondents by sex, socioeconomic status, and race and ethnicity^44^; however, data on race and ethnicity were not available for this cohort.

### Statistical Analysis

The association between childhood symptom profiles and outcomes was assessed using tobit regression for earnings and negative binomial regression models for welfare receipt, intimate partnership, and children in the household, with adjustment for control variables including cohort type (representative vs disruptive sample). This was conducted in 2 steps. First, we examined the association with the outcomes for the high externalizing, high internalizing, and comorbid symptoms profiles, with the no/low symptoms profile treated as the reference category. Second, we repeated the analyses using the high externalizing profile and then the high internalizing profile as the reference category, so that the comorbid symptoms profile could be compared with the high externalizing and high internalizing symptoms profiles. To test whether the association between symptom profiles and outcomes differed for males and females, we systematically entered the child’s sex-by-profile interaction terms.

To examine the lost earnings associated with a 40-year working career, the following calculation was made:
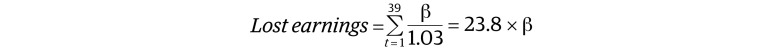
where β is the estimate obtained from the fully adjusted regression model, assuming an annual discount rate of 3% per year. The lost earnings calculation reflects the amount of the annual individual earnings that would be lost for participants in (1) the high externalizing profile, (2) high internalizing profile, and (3) comorbid profile, compared with the low-symptom profile. Missing data were managed using multiple imputations by chained equations.^45^ Results were pooled and analyses conducted across 50 data sets. Factors associated with missingness in the main outcome (earnings: 159 [5.3%]) were male sex and being in the comorbid group (eTable 2 in Supplement 1). All analyses were conducted using Stata, version 14.2 (StataCorp LLC). The significance level was set at *P* < .05, and all tests were 2-tailed.

## Results

Of 3017 participants in this sample, 1594 (52.8%) were male and 1423 (47.2%) were female. Per confidentiality rules established by Statistics Canada, income variables were rounded to base 100 and count variables were rounded to base 10. Mean (SD) age at follow-up was 37 (0.29) years. Participants’ childhood and family characteristics, stratified by symptom profile, are shown in Table 1. Participants in the sample earned a mean (SD) of $32 800 ($26 000) per year at age 33 to 37 years (2013-2017). Across the 20 years of follow-up, participants received welfare support for a mean (SD) of 1.45 (3.53) years, had an intimate partner for 7.37 (5.20) years, and had children living in the household for 11 (5.40) years. Participants in the comorbid symptom profile were more likely to be male vs female (82.3% vs 17.7%), to have younger mothers, to come from households with lower earnings when they were aged 3 to 5 years, and to have a nonintact family at age 6 years.

**Table 1.** Sample Characteristics at Baseline and Follow-up Stratified by Behavioral Problem Profile^a^

Characteristic	Total (N = 3017), mean (SD) [range]	Mean (SD)			
		No/low symptoms (n = 1369])	Externalizing symptoms (n = 882)	Internalizing symptoms (n = 354)	Comorbid symptoms (n = 412)
Childhood					
Male, No. (%)	1594 (52.8)	545 (39.8)	566 (64.2)	144 (40.7)	339 (82.2)
Female, No. (%)	1423 (47.2)	824 (60.2)	316 (35.8)	210 (59.3)	73 (17.7)
Verbal IQ	9.82 (1.54) [1-13]	10.05 (1.36)	9.81 (1.50)	9.50 (1.55)	9.20 (2.11)
Mother’s age at birth of first child, y	24.31 (3.92) [13.63-43.41]	24.68 (3.77)	24.11 (4.00)	24.38 (4.08)	23.29 (4.01)
Father’s age at birth of first child, y	26.76 (4.12) [13.90-49.24]	26.85 (3.93)	26.74 (4.34)	26.83 (4.09)	26.34 (4.37)
Mother’s years of education	11.70 (2.63) [0-22]	12.12 (2.59)	11.53 (2.63)	11.34 (2.68)	11.03 (2.50)
Father’s years of education	11.84 (3.42) [0-30]	12.35 (3.51)	11.61 (3.36)	11.46 (3.24)	10.91 (3.05)
Parents’ household income, $	24 600 (14 900) [400-159 300]	27 500 (14 000)	23 300 (15 800)	23 800 (14 800)	18 500 (13 200)
Intact family structure, No. (%)	1780 (83.4)	940 (90.2)	490 (80.7)	220 (82.8)	140 (60.3)
Disruptive cohort, No. (%)^b^	1020 (33.8)	309 (22.6)	378 (42.9)	100 (28.2)	238 (57.6)
Adulthood					
Employment earnings, age 33-37 y, $	32 800 (26 000) [0-199 900]	37 300 (26 900)	32 700 (26 200)	26 500 (20 700)	23 100 (23 100)
Year of welfare receipt, age 18-37 y	1.45 (3.53) [0-20]	0.68 (2.41)	1.56 (3.50)	1.72 (3.85)	3.58 (5.19)
Years with intimate partner, age 18-37 y	7.37 (5.20) [0-20]	8.13 (5.11)	7.12 (5.22)	7.20 (5.21)	5.32 (4.87)
Years of children living in household, age 18-37 y	11.09 (5.40) [0-20]	11.66 (5.06)	11.11 (5.56)	10.98 (5.39)	9.18 (5.75)

^a^ Up to 3.1% missing data except for intact family, which had 29.2% missing. In accordance with Statistics Canada’s confidentiality (nondisclosure) rules, earnings are rounded to the nearest hundred, and ranges represent the mean of the 5 lowest and 5 highest scores and are therefore a conservative estimate of the upper limit. Personal earnings and household income represent the annual mean for the 2013-2017 period. Note that welfare, intimate partnership (ie, marriage or cohabitation lasting ≥1 year), and children variables represent count data. Scores therefore indicate the mean number of years in which participants reported the relevant item in their tax return.

^b^ The disruptive cohort comprised children who scored at or above the 80th percentile for disruptive behavior problems at the end of kindergarten (age 5 or 6 years) using sex-specific cutoffs.

**Figure 2.**
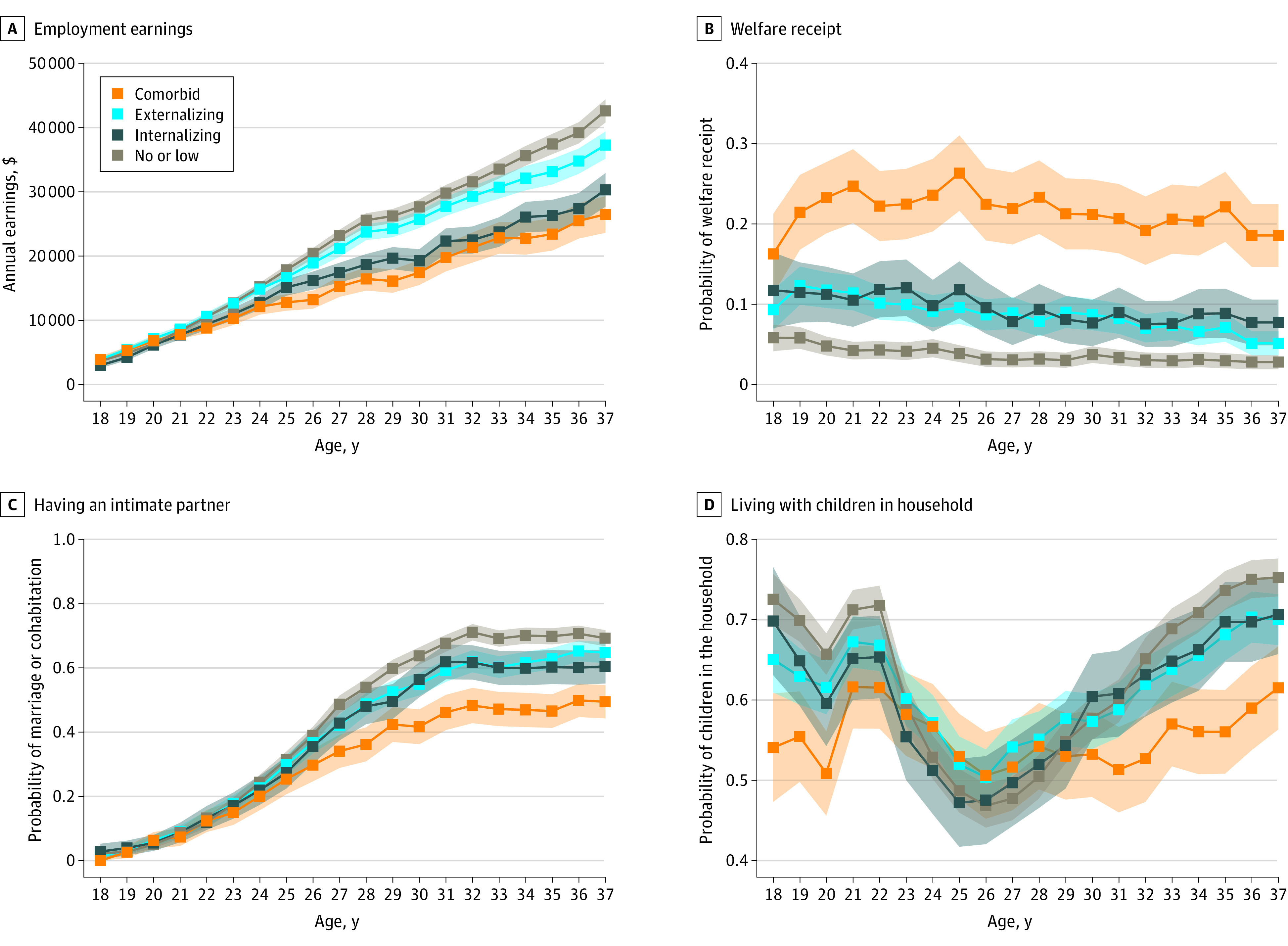
Descriptive Statistics for Economic and Social Outcomes From Age 18 to 37 Years, According to Childhood Symptom Profile Participants with no/low symptoms (1369 [45.4%), high externalizing symptoms (882 [29.2%]), internalizing symptoms (354 [11.7%]), and comorbid symptoms (412 [13.7%]). Data points show means with SEs in shading.

Figure 2 shows participants’ employment earnings, years of welfare receipt, years intimately partnered, and years of children living in the household. Participants in the high externalizing and high internalizing symptom profiles and, especially, the comorbid profile, had lower earnings and a higher incidence of annual welfare receipt across early adulthood and were less likely to have an intimate partner and to have children living in the household, compared with participants in the no/low symptoms profile.

Multivariable analyses showed that, compared with the no/low symptom profile, participants in the high externalizing, high internalizing, and comorbid profiles had worse economic and social outcomes, with the latter profile faring particularly poorly (Table 2). Participants in the childhood comorbid symptom profile earned $15 031 (95% CI, −$18 030 to −$12 031) less per year, had a 3.79 (95% CI, 2.75-5.23) times higher incidence of annual welfare receipt across follow-up, and were less likely to likely to be married or cohabiting with an intimate partner (incident rate ratio [IRR], 0.71; 95% CI, 0.63-0.79) and to have children living in the household (IRR, 0.86; 95% CI, 0.80-0.92).

**Table 2.** Association Between Child Longitudinal Symptom Profiles and Adult Life Economic and Social Outcomes^a^

Characteristic	Personal earnings, aged 33-37 y, β coefficient (95% CI), $^b^	IRR (95% CI)		
		Welfare receipt, aged 19-37 y	Intimate partner, aged 19-37 y	Children in household, aged 19-37 y
**No/low symptoms**				
Externalizing	−5904 (−7988 to −3821)^c^	2.0 (1.58 to 2.53)^c^	0.93 (0.86 to 1.01)	0.99 (0.94 to 1.04)
Internalizing	−8473 (−11 228 to −5717)^c^	2.07 (1.51 to 2.83)^c^	0.89 (0.80 to 0.99)^d^	0.95 (0.88 to 1.01)
Comorbid	−15 031 (−18 030 to −12 031)^c^	3.79 (2.75 to 5.23)^c^	0.71 (0.63 to 0.79)^c^	0.86 (0.80 to 0.92)^c^
Sex (male)^e^	13 105 (11 357 to 14 852)^c^	0.74 (0.61 to 0.91)^c^	0.80 (0.75 to 0.86)^c^	0.88 (0.84 to 0.92)^c^
**Externalizing symptoms**				
No symptoms	5904 (3821 to 7988)	0.50 (0.40 to 0.63)^c^	1.08 (0.99 to 1.17)	1.01 (0.96 to 1.06)
Internalizing	−2568 (−5475 to 338)^c^	1.03 (0.75 to 1.43)	0.96 (0.86 to 1.07)	0.96 (0.89 to 0.03)
Comorbid	−9126 (−12 030 to −6222)^c^	1.90 (1.38 to 2.61)^c^	0.76 (0.68 to 0.85)^c^	0.87 (0.81 to 0.93)^c^
**Internalizing symptoms**				
No symptoms	8473 (5717 to 11 228)^c^	0.48 (0.35 to 0.66)^c^	1.12 (1.01 to 1.25)^c^	1.06 (0.99 to 1.13)
Externalizing	2568 (−339 to 5474)	0.97 (0.70 to 1.34)	1.04 (0.93 to 1.16)	1.05 (0.97 to 1.13)
Comorbid	−6558 (−10 104 to −3011)^c^	1.84 (1.26 to 2.68)^c^	0.79 (0.69 to 0.91)^c^	0.91 (0.83 to 0.99)^d^

Abbreviations: IRR, incident rate ratio.

^a^ All models adjusted for sex, child IQ, parents’ age at birth of first child, parents’ years of education, parents’ household income, family structure, the child’s relative age in the classroom, and cohort type.

^b^ Unstandardized β coefficient.

^c^ *P* < .01.

^d^ *P* < .05.

^e^ Male was the reference category.

Across the sample, males had higher mean annual employment earnings ($13 105; 95% CI, $11 357-$14 852) and were less likely to receive welfare each year (IRR, 0.74; 95% CI, 0.61-0.91) but were less likely to have an intimate partner (IRR, 0.80; 95% CI, 0.75-0.86) and to have children living in the household (IRR, 0.88; 95% CI, 0.84-0.92) compared with females. There were 2 significant sex-by-trajectory profile membership interactions. Compared with females in the high externalizing profile, males in the high externalizing profile were significantly less likely to be in receipt of welfare (IRR, 0.50; 95% CI, 0.31-0.80; *P* = .004), and males in the comorbid profile were less likely to have children living in the household (IRR, 0.80; 95% CI, 0.69-0.94) compared with females in the comorbid profile.

Compared with the high externalizing profile, participants in the comorbid profile still fared significantly worse: they earned $9126 less per year (95% CI, −$12 030 to −$6222), had a higher incidence of annual welfare receipt (IRR, 1.90; 95% CI, 1.38-2.61), had a lower incidence of intimate partnership (IRR, 0.76; 95% CI, 0.68-0.85), and were less likely to have children living in the household (IRR, 0.87; 95% CI, 0.81-0.93). Participants in the comorbid symptoms profile also fared poorly compared with the high internalizing profile, earning $6558 less per year (95% CI, −$10 104 to −$3011), having a higher incidence of welfare receipt (IRR, 1.84; 95% CI, 1.26-2.68), and having a lower incidence of intimate partnership (IRR, 0.79; 95% CI, 0.69-0.91) and children living in the household (IRR, 0.91; 95% CI, 0.83-0.99) (Table 2). Participants in the high internalizing profile also earned $2568 less per year compared with participants in the high externalizing profile. Compared with the no/low symptom profile, participants in the high externalizing-only profile earned $5904 (95% CI, −$7988 to −$3821) less per year and had 2.0 (95% CI, 1.58-2.53) times higher incidence of welfare receipt, while participants in the high internalizing group earned $8473 (95% CI, −$11 228 to −$5717) less per year, had a 2.07 (95% CI, 1.51-2.83) higher incidence of welfare receipt, and a lower incidence of intimate partnership (incident rate ratio [IRR], 0.89; 95% CI, 0.80-0.99). There were no significant interactions between the high externalizing-only, high internalizing-only, and comorbid symptoms profile comparisons. Over a 40-year working career, estimated lost personal employment earnings were $140 515 for the high externalizing profile, $201 657 for the high internalizing profile, and $357 737 for the comorbid profile, compared with participants in the no/low symptom profile. Results from the nonimputed analysis showed smaller estimates and no significant interactions, but otherwise did not substantively differ from the main results, supporting the robustness of the findings (eTable 3 in Supplement 1).

## Discussion

In this large Canadian population-based sample, children who exhibited consistently high externalizing symptoms, high internalizing symptoms, or comorbid high externalizing and high internalizing symptoms had poor long-term economic and social outcomes across early adulthood compared with children without behavioral symptoms. Children with comorbid high externalizing and high internalizing symptoms fared particularly poorly: their average annual employment earnings were nearly 2 times lower than participants who had childhood high internalizing problems only and nearly 3 times lower than participants who had high externalizing problems only. They were also significantly more likely to have no intimate partner and to live without children in the household into middle age. Compared with those with no/low symptoms, children who exhibited comorbid symptoms experienced substantial economic losses estimated at $357 737 in lost earnings across their working lives.

Our results build on a large body of literature linking childhood high externalizing symptoms and high internalizing symptoms to adverse social and economic outcomes across the life course.^6,7,8,46,47,48^ Compared with the no/low symptom profile, participants in the high internalizing profile earned substantially less than participants in the high externalizing profile ($5904 vs $8473), even after adjusting for sex differences in earnings, which may be attributable to the higher burden of major depressive disorder in this population. For children in the high externalizing profile, estimated lost earnings across their working lives amounted to more than $140 515, while for children in the high externalizing profile the difference was more than $201 657. But it was children with comorbid symptoms who appeared to be most vulnerable across all economic and social outcomes, and additional resources to support these children are required.

Several features of the symptom trajectories should be highlighted. First, consistent with prior research, males were more likely to exhibit high externalizing symptoms, while females were more likely to exhibit high internalizing symptoms.^49^ Second, 82.3% of participants in the comorbid group were male. This pattern concurs with at least 1 previous study,^50^ although others report a higher prevalence among females^14,15,16^ or no differences.^13^ Third, the mean high externalizing symptom severity level for participants in the comorbid group was higher than the mean symptom severity level in the high externalizing-only profile (Figure 1). This raises the possibility that the poorer outcomes in the comorbid group were in fact attributable to the overall higher levels of high externalizing symptoms, rather than to comorbidity per se—something that cannot be ruled out in the current study.

### Explanatory Pathways

Several mechanisms could explain the associations observed in this study. According to Moffit’s^51^ snare hypothesis, children with early behavior problems are more likely to become trapped in risky adolescent activities, such as substance use, delinquent peer affiliations, and academic underachievement, which hampers their transition to adulthood and undermines the accumulation of social and economic capital across the life course.^52^ For children with comorbid symptoms, the effect on long-term outcomes is likely to be compounded as multiple domains of functioning are impacted across development. Indeed, children with comorbid behavior problems are more likely to experience a range of psychosocial, health, and education difficulties across childhood and adolescence, including teacher conflict and lower educational level attainment,^18^ delinquency,^23^ substance use problems,^21,22^ mental disorders and suicidal behavior,^11,19^ and higher health service use.^53^ These events can be especially harmful to economic participation, as they undermine education and occupational attainment and performance and have knock-on effects on social functioning, including intimate partnership formation, through negative reinforcing cycles.^11,54,55^

### Implications for Prevention

Children with elevated comorbid symptoms are highly vulnerable to poor adult-life outcomes that are robustly associated with health, well-being, and longevity.^29,56,57,58^ Early detection, prevention, and support are critical. Very little is currently known about how to prevent the development of multiple comorbid disorders, and efforts should focus on prevention and support for specific categories of disorders. For externalizing problems, programs to reduce inattentive and disruptive behaviors and promote prosocial and socioemotional skills are effective,^59,60,61,62,63^ especially for children from disadvantaged backgrounds.^64^ Reducing childhood anxiety and depressive symptoms is more challenging, however, and meta-analyses show small or weak effect sizes.^65,66,67^ Since internalizing problems may increase due to externalizing problems,^68^ programs that specifically address internalizing problems in children with already-elevated externalizing problems may also be relevant. More work is needed to develop and test interventions for children with comorbid symptoms,^69,70^ which includes clarifying the additive vs interactive relationship between externalizing and internalizing symptoms and improving understanding of the mechanisms that underlie the observed associations as the basis for prevention planning.

### Strengths and Limitations

The strengths of this study were its 32-year follow-up duration, the use of teacher-rated behavioral assessments across 7 years of childhood, and the testing of multiple domains of economic and social outcomes obtained from administrative data (tax records). This is also the first study, to our knowledge, to examine long-term outcomes for comorbid symptoms with reference to high externalizing symptoms or high internalizing symptoms only.

This study also has limitations. First, this was an observational study; many intervening life events on the pathway to adult life (eg, substance use, academic underachievement) could account for the observed associations and should be tested in future studies. Second, some studies indicate that external observers, such as teachers, are not best placed to assess internalizing symptoms in children, and this could have led to an underestimation of symptom severity in this domain. Third, unmeasured confounders, such as parental mental health, could also have influenced outcomes. Fourth, the lost lifetime earnings calculation is based on projected estimates obtained from a 5-year earnings average, which may not have accounted for all sources of variance (eg, parental leave, unemployment), and should be understood as approximate only.

## Conclusions

The findings of this cohort study suggest that children who exhibit increased externalizing or internalizing symptoms across childhood are at risk of poor economic and social outcomes in adulthood. Comorbid externalizing and internalizing symptom presentations indicated especially high risk. Improving early identification and prevention for children with comorbid symptoms is essential and may have large benefits for both individuals and society.

## Notes

Supplement 1. **eTable 1.** Measurement Invariance for the Behavioral Symptom Trajectories **eTable 2.** Missing Data Patterns **eTable 3.** Multivariable Models of Association Between Child Longitudinal Symptom Profiles and Adult Life Economic and Social Outcomes for the Nonimputed Sample

Supplement 2.
Data sharing statement
