# Supplementary material for: Association of Childhood Externalizing, Internalizing, and Comorbid Symptoms With Long-term Economic and Social Outcomes
Source: JAMA Netw Open. 2023 Jan 9;6(1):e2249568. doi: 10.1001/jamanetworkopen.2022.49568 (PMC9856729; doi:10.1001/jamanetworkopen.2022.49568)
Supplement: Supplement 1. — eTable 1. Measurement Invariance for the Behavioral Symptom Trajectories eTable 2. Missing Data Patterns eTable 3. Multivariable Models of Association Between Child Longitudinal Symptom Profiles and Adult Life Economic and Social Outcomes for the Nonimputed Sample [file jamanetwopen-e2249568-s001.pdf]

Supplementary Online Content

Vergunst F, Commisso M, Geoffroy MC, et al. Association of childhood externalizing, internalizing, and comorbid symptoms with long-term economic and social outcomes. *JAMA Netw Open*. 2023;6(1):e2249568. doi:10.1001/jamanetworkopen.2022.49568

eTable 1. Measurement Invariance for the Behavioral Symptom Trajectories

eTable 2. Missing Data Patterns

eTable 3. Multivariable Models of Association Between Child Longitudinal Symptom Profiles and Adult Life Economic and Social Outcomes for the Nonimputed Sample

This supplementary material has been provided by the authors to give readers additional information about their work.

© 2023 Vergunst F et al. *JAMA Network Open.*

eTable 1. Measurement Invariance for the Behavioral Symptom Trajectories

**Measurement invariance model**	**$\chi2$(df)**	**CFI**	**RMSEA**	**ΔCFI**	**ΔRMSEA**
**Externalizing factor**					
Configural (no equality constraints)	7783.2 (1255)	0.823	0.063		
Metric (loadings)	8597.3 (1291)	0.802	0.066	0.023	0.003
Scalar (loadings + intercepts)	10161.9 (1327)	0.761	0.071	0.041	0.006
**Internalizing factor**					
Configural (no equality constraints)	1160.7 (159)	0.879	0.062		
Metric (loadings)	1253.4 (171)	0.869	0.063	0.010	0.000
Scalar (loadings + intercepts)	1299.0 (183)	0.865	0.061	0.004	0.001

Note. df=degrees of freedom, CFI=comparative fit index, RMSEA=Root Mean Square Error Approximation, Δ=change in value.

© 2023 Vergunst F et al. *JAMA Network Open.*

eTable 2 Missing data patterns

	N	(%)
**Predictor and outcome**		
Symptom profiles, age 6-12 years	<5	(<1%)
Sex	<5	(<1%)
Verbal IQ	1310	(43.3%)
Mother's age at birth of first child	422	(14.0%)
Father's age at birth of first child	630	(21.0%)
Mother's years of education	100	(3.4%)
Father's years of education	350	(11.4%)
Parents' household income	60	(2.0%)
Intact family structure	880	(29.2%)
Annual employment earnings, age 33-37 years	140	(4.6%)

Note. Predictors of missingness in the outcome (n=159) were male sex (p=.001) and being in the comorbid group (p=.001). Other predictors were not significantly associated with missingness. Counts are rounded to base ten and scores below 5 are suppressed due to Statistics Canada data protection requirements. Missing data were handled using multiple imputations by chained equations. Results were pooled and analyses conducted across 50 datasets.

© 2023 Vergunst F et al. *JAMA Network Open.*

eTable 3 Multivariable models of association between child longitudinal symptom profiles and adult life economic and social outcomes for the non-imputed sample (n=1260)

	Personal earnings, age 33-37 years		Welfare receipt, age 19-37 years		Married/cohabiting, age 19-37 years		Children in household, age 19-37 years	
	b	95%CI	IRR	95%CI	IRR	95%CI	IRR	95%CI
(No symptoms)								
Externalising	-6016	-9326 – -2706**	2.06	1.38 – 3.07 **	0.93	0.83 – 1.04	0.95	0.90 1.01
Internalising	-8235	-12437 – -4032**	2.16	1.29 – 3.61 **	0.92	0.79 – 1.06	0.96	0.89 – 1.04
Co-morbid	-14470	-19793 – -9147**	3.30	1.81 – 6.06 **	0.75	0.62 – 0.91 **	0.87	0.80 – 0.96 **
Sex (m)	13344	10577 – 16110**	0.83	0.59 – 1.17 **	0.79	0.71 – 0.86 **	0.91	0.86 – 0.96 **
(Externalising)								
No symptoms	5817	2658 – 8976 **	0.49	0.33 – 0.73 **	1.08	0.96 – 1.21	1.05	0.99 – 1.11
Internalising	-2122	-6535 – 2291	1.05	0.61 – 1.80	0.98	0.84 – 1.16	1.01	0.93 – 1.10
Co-morbid	-7998	-13060 – -2937 **	1.61	0.86 – 3.0	0.81	0.67 – 0.98 *	0.92	0.99 – 1.11†
(Internalising)								
No symptoms	7939	3934 – 11944 **	0.46	0.28 – 0.77 **	1.09	0.94 – 1.26	1.04	0.96 – 1.12
Externalising	2122	-2291 – 6536	0.95	0.55 – 1.63	1.01	0.86 – 1.19	0.99	0.91 – 1.08
Co-morbid	-5876	-11739 – -13 *	1.53	0.76 – 3.07	0.82	0.66 – 1.02 †	0.91	0.81 – 1.12 †

Note. b=unstandardised beta coefficient, IRR=incident rate ratio. Reference group is shown in parentheses. All models adjusted for sex, child IQ, parents' age at birth of first child, parents' years of education, parents' household income, family structure, the child's relative age in the classroom, and cohort type. ** p<0.01. * p<0.05. † p<0.10

© 2023 Vergunst F et al. *JAMA Network Open.*
