# Supplementary material for: Association of Childhood Externalizing, Internalizing, and Comorbid Symptoms With Long-term Economic and Social Outcomes
Source: JAMA Netw Open. 2023 Jan 9;6(1):e2249568. doi: 10.1001/jamanetworkopen.2022.49568 (PMC9856729; doi:10.1001/jamanetworkopen.2022.49568)
Supplement: Supplement 2. — Data sharing statement [file jamanetwopen-e2249568-s002.pdf]

Data Sharing Statement

Vergunst. Association of Childhood Externalizing, Internalizing, and Comorbid Symptoms With Long-term Economic and Social Outcomes. *JAMA Netw Open*. Published January 09, 2023. doi:10.1001/jamanetworkopen.2022.49568

Data
Data available: No

Additional Information
Explanation for why data not available: Data are available by application.
